# Sex Hormone Binding Globulin Modifies Testosterone Action and Metabolism in Prostate Cancer Cells

**DOI:** 10.1155/2016/6437585

**Published:** 2016-11-20

**Authors:** Huika Li, Thy Pham, Brett C. McWhinney, Jacobus P. Ungerer, Carel J. Pretorius, Derek J. Richard, Robin H. Mortimer, Michael C. d'Emden, Kerry Richard

**Affiliations:** ^1^Conjoint Endocrine Laboratory, Chemical Pathology, Pathology Queensland, Queensland Health, Herston, QLD 4029, Australia; ^2^School of Biomedical Sciences, Queensland University of Technology, Brisbane, QLD 4000, Australia; ^3^Department of Endocrinology and Diabetes, Royal Brisbane and Women's Hospital, Herston, QLD 4029, Australia

## Abstract

Sex Hormone Binding Globulin (SHBG) is the major serum carrier of sex hormones. However, growing evidence suggests that SHBG is internalised and plays a role in regulating intracellular hormone action. This study was to determine whether SHBG plays a role in testosterone uptake, metabolism, and action in the androgen sensitive LNCaP prostate cancer cell line. Internalisation of SHBG and testosterone, the effects of SHBG on testosterone uptake, metabolism, regulation of androgen responsive genes, and cell growth were assessed. LNCaP cells internalised SHBG by a testosterone independent process. Testosterone was rapidly taken up and effluxed as testosterone-glucuronide; however this effect was reduced by the presence of SHBG. Addition of SHBG, rather than reducing testosterone bioavailability, further increased testosterone-induced expression of prostate specific antigen and enhanced testosterone-induced reduction of androgen receptor mRNA expression. Following 38 hours of testosterone treatment cell morphology changed and growth declined; however, cotreatment with SHBG abrogated these inhibitory effects. These findings clearly demonstrate that internalised SHBG plays an important regulatory and intracellular role in modifying testosterone action and this has important implications for the role of SHBG in health and disease.

## 1. Introduction

Sex Hormone Binding Globulin (SHBG) is the major sex hormone carrier protein in serum. Under physiological conditions, approximately 70% of testosterone (Te) is bound to SHBG with high affinity, about 20–30% is weakly bound to albumin, and the remaining 1-2% is free [1, 2]. Pubertal increases in serum testosterone in males peak at 20 years of age and remain stable until the eighth decade. After childhood, SHBG declines by the age of 20 years and then remains stable until the sixth decade with a gradual, progressive rise thereafter [3].

For many years it has been widely accepted that the primary function of SHBG is to transport sex steroid hormones to target tissues where SHBG modulates the level of free sex hormones that can enter target cells [4, 5]. However, in recent years, additional roles for SHBG have been identified. There is strong evidence that SHBG mediates steroid hormone signal transduction at the plasma membrane [6] and a growing body of evidence that SHBG may be taken up by cells and have intracellular biological functions [7]. A study has demonstrated that SHBG is internalised through the Megalin receptor in rat yolk sac cells and that Megalin deficient mice display defects similar to animals treated with androgen- or estrogen-receptor antagonists [4]. SHBG internalisation has also been described in other cell types such as periventricular neurons and fibroblast cells, suggesting that it may have an intracellular role [8, 9]. Intracellular expression of SHBG in mouse proximal tubule cells has been demonstrated to increase uptake of ^3^H-dihydrotestosterone (DHT) and to prolong the expression of androgen responsive genes [10].

This study was designed to determine whether the androgen sensitive LNCaP prostate cancer cell line [11] internalises SHBG; whether SHBG uptake affects Te uptake; the effects of SHBG on Te function and metabolism; and the effects of SHBG and Te on cell growth.

## 2. Materials and Methods

### 2.1. Cell Culture

The androgen responsive human prostate cancer cell line LNCaP clone FGC (ATCC, VA, USA) was chosen for this study. Cells were cultured in Roswell Park Memorial Institute (RPMI) medium (Sigma-Aldrich, VIC, Australia) supplemented with 10% fetal bovine serum (FBS), 500 U/mL penicillin streptomycin (Gibco, VIC, Australia), and 5 *μ*g/mL plasmocin (InvivoGen, VIC, Australia) in a conventional cell culture incubator. All experiments were conducted in serum-free medium to eliminate SHBG and albumin contamination from FBS and in the absence of phenol red, which can bind to steroid receptors [12].

### 2.2. Reagents

Purified native human SHBG protein was purchased from Fitzgerald Industries International, MA, USA. SHBG was purified from the serum of pregnant women by ligand affinity chromatography with a purity of >98% with an unspecified residual steroid content. Testosterone was purchased from Sigma-Aldrich, VIC, Australia, and prepared as a 500 mM stock in ethanol. Testosterone was diluted >5 × 10^6^ times in serum-free medium (final < 0.00002% ethanol) and used at a maximal final concentration of 100 nM in experiments. Ethanol had no effects on LNCaP cell growth at concentrations between 0.00001% and 1% over 96 hours (data not shown).

Unless specified all other reagents were purchased from Sigma-Aldrich.

### 2.3. Alexa Fluor® 546 SHBG Labeling and Internalisation

Purified native human SHBG protein was labeled using an Alexa Fluor 546 Protein Labeling Kit (Life Technologies, VIC, Australia). Briefly, 1 mL of 2 mg/mL purified SHBG protein was incubated with Alexa Fluor 546 reactive dye for 2 hours at room temperature. Then Alexa Fluor 546 labeled-SHBG (Alexa546-SHBG) was purified through a Bio-Gel P-6 Gel column (BioRad, CA, USA). To determine the concentration of labeled SHBG and the degree of fluorescent labeling, the conjugate solution was measured at 280 nm and 540 nm in a Nanodrop 2000 spectrophotometer (Thermo Scientific, NC, USA).

For Alexa546-SHBG uptake experiments, the cells were serum-starved for 1 hour and then incubated in 125 nM Alexa546-SHBG ± 25 nM Te in serum-free RPMI for 1 hour at room temperature. Cells were washed with Phosphate Buffered Saline (PBS), fixed in 2% paraformaldehyde, and permeabilised in 0.2% Triton X-100 in PBS. Cells were costained with Alexa488-phalloidin to highlight the *β*-actin skeleton and nuclei were stained with DAPI. Coverslips were mounted with Prolong® Gold Antifade reagent. Images were captured using a DeltaVision Deconvolution Microscope (Applied Precision, Washington DC, USA). Uptake of Alexa546-SHBG was confirmed by costaining internalised Alexa546-SHBG with a monoclonal anti-SHBG antibody (Sigma, VIC, Australia) conjugated to an Alexa488 labeled secondary antibody. The colocalisation of Alexa546 (red) and Alexa488 (green) discriminated Alexa-SHBG from endogenous SHBG, which was stained with Alexa488 only. Treatments with serum-free medium only and Alexa546 dye only were used as negative controls.

SHBG secretion from LNCaP cells was not detectable (data not shown) by Western blot or Unicel® immunoassay (lower limit of detection < 2 nM), despite cell culture medium being concentrated 12-fold; therefore endogenous levels of SHBG should have little effect on our experiments using 125 nM SHBG.

### 2.4. Measurement of Te Uptake by Tandem Mass Spectrometry

Cells were cultured in 6-well plates and serum-starved for 4 hours prior to uptake experiments. Cells were then incubated in serum-free uptake medium containing 25 nM (30 pmoles/well) of Te ± 125 nM SHBG for 1, 4, and 24 hours. Control cells were in culture medium only. At the end of each time point the culture medium was collected for Te measurements. Cell monolayers were washed carefully with 2 mL PBS (pH 7.3) and then cells were lysed with Pierce M-PER Mammalian Protein Extraction Reagent (Thermo Fisher Scientific) to extract soluble protein. Further lysis with Pierce RIPA Lysis and Extraction Buffer (Thermo Fisher Scientific) was used to extract remaining proteins from the membrane and nuclei. Te was measured in the original uptake medium (25 nM), cell culture medium, and cell lysates using Tandem Mass Spectrometry. Te measured in all fractions was expressed as a percentage of the Te in the original uptake medium. Cellular Te includes all Te measured in both lysates including cytosol, membrane, and nuclei. Total Te includes all Te measured in cellular fractions and cell culture medium.

Liquid Chromatography followed by Tandem Mass Spectrometry (LC MSMS) was used to measure Te on a Waters™ Ultra Performance Liquid Chromatography (UPLC) Acquity system. Briefly, samples (in 2 mL 96 deep well plates) were precipitated with 3 volume equivalents of 50 mM zinc sulphate/40% methanol containing an internal standard (testosterone d2). After protein precipitation, the samples were centrifuged and the plate was transferred to an autosampler. An aliquot of 50 *μ*L was injected onto a Waters Online Sample Manager (OSM). The extract was concentrated onto the MassTrak XBridge C18 OSM 10 *μ*m cartridge, washed with 30% methanol, and then switched into line and eluted directly into the UPLC system. The analytes were separated on an Acquity BEH C_18_ 2.1 × 50 mm column. The column eluent was directed (without stream splitting) into the ion source of a Waters Xevo TQ-D tandem quadrupole MS, operated in positive ESI mode. The following quantifier and qualifier transitions were utilised: testosterone (289.1 > 97.0, 289.1 > 109.0) and testosterone d2 (291.1 > 111.0). The gradient was returned to initial conditions in preparation for the next sample, which is being prepared in parallel by the OSM. Cycle time between samples is 3 min. The limit of the blank was 0.04 nmol/L with an interrun imprecision of 9.8% for testosterone at 0.9 nmol/L.

### 2.5. Deconjugation of Te-Glucuronide

Measurement of Te-glucuronide formation and efflux into medium was determined by digestion of cell culture medium with* E. coli β*-glucuronidase (Sigma, VIC, Australia), followed by measurement of Te by LC MSMS. Enzymatic deconjugation of Te-glucuronide was optimised by using two different concentrations of *β*-glucuronidase (200 U/mL and 400 U/mL), two different dilutions of sample (culture medium neat and diluted 1 : 10) for 24 hours incubation time. Maximal deconjugation was achieved in neat samples incubated with 200 U/mL *β*-glucuronidase for 24 hours at 37°C. Te-glucuronide was not detectable in cell lysates; therefore only effluxed Te-glucuronide in cell culture medium was measured.

After culturing cells with 25 nM (30 pmoles/well) Te ± 125 nM SHBG for 4 or 24 hours, culture medium was diluted in an equal volume of 0.25 M potassium phosphate buffer, pH 6.9 ± 200 units/mL of* E. coli β*-glucuronidase (Sigma, VIC, Australia) and incubated at 37°C for 24 hours [13]. To quantitate the amount of Te-glucuronide, two sets of identical samples were prepared with or without *β*-glucuronidase. The quantity of Te in each sample was measured by LC MSMS and expressed as a percentage of Te in the original uptake medium. Unconjugated Te was detectable before digestion with *β*-glucuronidase. Following treatment with *β*-glucuronidase both unconjugated and previously glucuronidated Te were measured; therefore the value for unconjugated Te was subtracted from this in order to quantify the glucuronidated fraction.

### 2.6. Quantitative Real-Time PCR (RT-PCR)

Total RNA was isolated using an RNeasy Mini Kit (QIAGEN, VIC, Australia). RNA concentrations and quality were determined using a NanoDrop-3000 Spectrophotometer (NanoDrop Technologies, Inc., NC, USA). Four micrograms of total RNA was reverse-transcribed with 200 units of Superscript III Reverse Transcriptase (Invitrogen Life Technologies, CA, USA) and 0.5 *μ*g of oligo (dT) 15 primers (Roche Diagnostics, Mannheim, Germany) in a reaction volume of 20 *μ*L. RT-PCR was performed using 3 *μ*L of cDNA (represented 6 ng RNA), 0.5 *μ*M of each primer (Table 1), and FastStart SYBR green PCR Master (Roche Diagnostics, Mannheim, Germany) in a Rotor Gene RG-6000 (Corbett Research, NSW, Australia). To quantify the gene expression profile in each sample, the efficiency of each standard curve was determined by its slope and comparative threshold. Data were presented as ratio of the amount of targeted mRNA (arbitrary units) normalised with the house-keeping gene beta 2-microglobulin (*β*2M)

### 2.7. Quantification of Alexa546-SHBG Uptake

LNCaP cells (~80,000 cells/well) were cultured on 24-well plates for 48 hours. Following serum starvation, cells were incubated with combinations of Alexa546-SHBG ± Te and imaged using an IncuCyte ZOOM™ Live Cell-Imaging System (Essen BioScience, MI, USA) with phase and red channels and scanned at 3-hour intervals for a total of 30 hours. At the end of the incubation period, cells were washed in an acid solution (200 mM glycine, 150 mM NaCl, pH 2.5) to remove surface bound Alexa546-SHBG [14]. IncuCyte ZOOM software was used to quantify the red objects in 9 nonoverlapping images per well. Data were presented as mean number of red objects per image.

### 2.8. Cell Growth Assay

LNCaP cells were cultured on 24-well plates with ~35,000 cells per well. After serum starvation the cells were treated with various combinations of Te ± SHBG and incubated in an IncuCyte ZOOM set to scan every 3 hours for up to 86 hours. The kinetic measurements of proliferation and data analysis were performed using IncuCyte ZOOM software according to manufacturer's instructions. Mean values of percentage confluence were calculated using 9 nonoverlapping images per well. The data was presented as percentage of phase object confluence.

### 2.9. Statistical Analysis

Statistical analysis was performed using GraphPad Prism Software (GraphPad Software, Inc., CA, USA). Comparison between groups was determined by one-way ANOVA with Tukey's test. *P* values of ≤0.05 were considered significant. All experiments were repeated at least three times on separate cell cultures and performed in triplicate for each treatment group.

## 3. Results

### 3.1. Internalisation of Alexa546-SHBG

**Figure 1:**
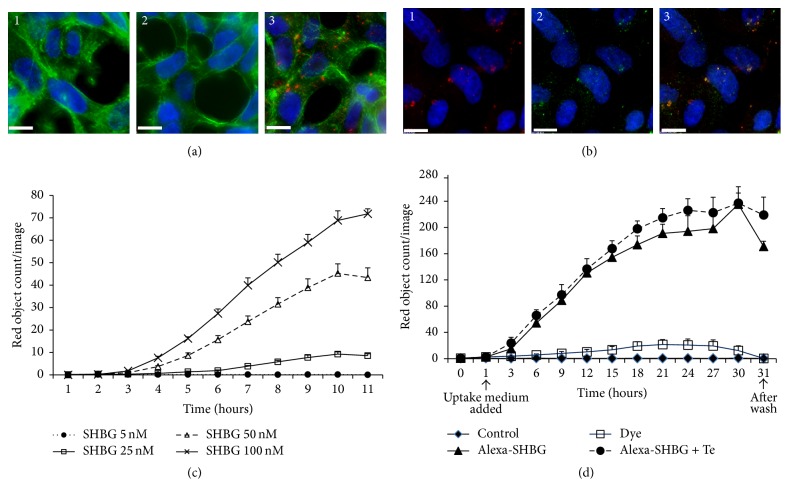
(a) Internalisation of Alexa Fluor 546 labeled SHBG (Alexa-SHBG) by LNCaP cells using a DeltaVision Deconvolution Microscope. Cells were incubated with (1) serum free RPMI medium, (2) Alexa 546 dye, and (3) Alexa Fluor 546 labeled SHBG (red) in serum-free medium for 1 hour and then costained with Alexa Fluor 488 phalloidin (green) to outline the actin cytoskeleton and DAPI (blue) to localise nuclei. Bar = 10 *μ*m. (b) To confirm Alexa labeled SHBG protein internalisation, cells were incubated with Alexa-SHBG for 1 hour and then costained with mouse anti-SHBG monoclonal antibody conjugated with anti-mouse Alexa Fluor 488 secondary antibody (green). (1) Internalised Alexa-SHBG (red) was detected inside the cells. (2) Intracellular SHBG was detected by anti-SHBG antibody (green). (3) Merged images (1) and (2) show internalised Alexa-SHBG in orange and endogenous SHBG in green. Bar = 10 *μ*m. (c) Alexa-SHBG uptake by LNCaP cells is dose dependent. The cells were incubated with 5 nM (closed circle), 25 nM (open square), 50 nM (open triangle), and 100 nM (cross) Alexa-SHBG for 30 hours. Data presented as mean number of red objects counted per image. Imaged using IncuCyte ZOOM Live Cell Imaging System. (d) Quantitation of Alexa-SHBG uptake by LNCaP cells using the IncuCyte ZOOM Live Cell Imaging System. Untreated LNCaP cells (control, closed diamond) and LNCaP cells treated with Alexa-546 dye only (dye, open square) were used as negative controls. Cells were starved with serum-free RPMI for 1 hour and treated with 50 nM Alexa546-SHBG only (closed triangle) and with 10 nM testosterone (Alexa-SHBG + Te, closed circle). Data presented as mean number of red objects counted per image.

Two methods were used to quantify Alexa546-SHBG uptake. Firstly, after incubation with Alexa546-SHBG, the cells were imaged using DeltaVision deconvolution microscopy (Figure 1(a)). Internalisation of Alexa546-SHBG was confirmed by generating an orthogonal view and a series of *z*-stack images. The red fluorescent signal representing Alex546 labeled SHBG was observed in the cells incubated with Alexa546-SHBG (Figure 1(a)(3)). There was no red signal observed in the negative controls incubated with unconjugated Alexa546 dye (Figure 1(a)(2)), confirming that Alexa-546 SHBG and not the Alexa dye itself was internalised by the cells. Additionally the internalised Alexa546-SHBG (red) colocalised with an Alexa488 (green) labeled anti-SHBG antibody (Figure 1(b)) further proving that SHBG was internalised while still being conjugated to Alexa546 dye. Endogenous SHBG is stained only with the Alexa488 anti-SHBG antibody (green). Secondly, to quantitate internalised Alexa546-SHBG IncuCyte ZOOM technology was used. A dose dependent uptake of Alexa546-SHBG was observed (Figure 1(c)). Addition of Te had no significant effect on Alexa-SHBG uptake (Figure 1(d)). Very weak signal was detected in Alexa546 dye treated culture and after the acid wash, no signal was detected.

### 3.2. Effect of SHBG on Te Uptake

**Figure 2:**
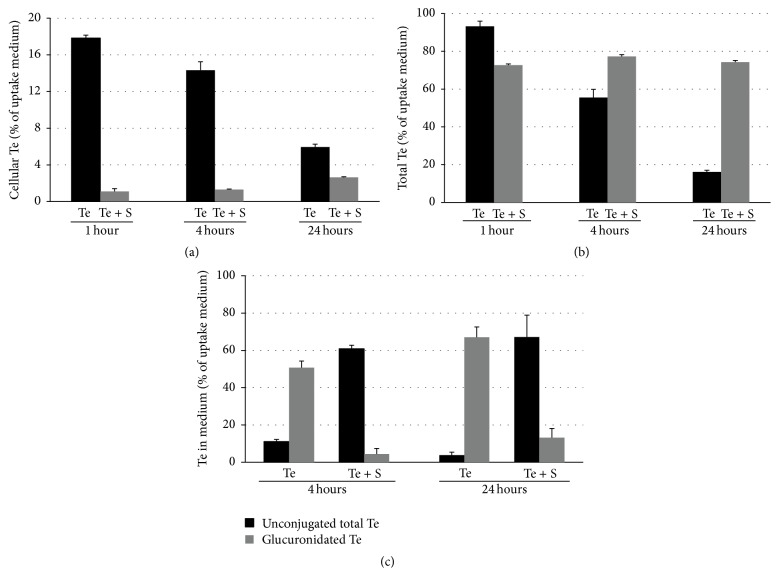
Effect of SHBG on testosterone uptake and metabolism by LNCaP cells measured by Liquid Chromatography/Tandem Mass (LC MSMS) Spectrometry. LNCaP cells were treated with 25 nM Te ± 125 nM SHBG (S) for 1, 4, and 24 hours. (a) Cellular Te and (b) total Te in culture medium and cell lysates (undigested). (c) Measurement of unconjugated (black) and glucuronidated (grey) Te in cell culture medium. Each medium sample was divided into two equal volumes and digested with or without 200 units/mL *β*-glucuronidase in potassium phosphate buffer (pH 6.9) for 24 hours at 37°C to revert glucuronidated testosterone to unconjugated testosterone. All measurements are expressed as a percentage of Te in the original uptake medium.

Incubation with 25 nM Te for 1, 4, and 24 hours resulted in rapid uptake of Te followed by a decline in cellular levels (Figure 2(a)). Cellular Te levels peaked at one hour (17.84 ± 0.31%) and then declined to 14.31 ± 0.93% and 5.95 ± 0.31% by 4 and 24 hours, respectively (Figure 2(a)). In contrast, in the presence of 125 nM SHBG, cellular Te levels were lower at 1 hour (1.10 ± 0.31%) and steadily increased to 1.32 ± 0.003% and 2.64 ± 0.04% by 4 and 24 hours. At 24 hours, cellular Te levels in cells incubated with Te plus SHBG were approximately half that of cells treated with Te alone.

Figure 2(b) shows the total Te measured in both the cell lysates and cell culture medium. When LNCaP cells were cultured in Te alone, 93 ± 2.80% of Te from the original uptake medium was measured at 1 hour but decreased to 55.50 ± 4.36% and then 16.79 ± 0.93% by 4 and 24 hours, respectively. When cells were cultured in Te plus SHBG, Te measurements did not change significantly over the three time points (72.69 ± 0.62%, 77.31 ± 0.93%, and 74.23 ± 0.93% at 1, 4, and 24 hours, resp.). Dihydrotestosterone was not detected in any of the samples.

### 3.3. Te-Glucuronide Formation

Cells were exposed to 25 nM Te ± 125 nM SHBG for 4 and 24 hours; then cell culture medium was collected and treated with *β*-glucuronidase. When LNCaP cells were cultured in Te for 4 hours, 50.73 ± 3.51% of Te was glucuronidated and effluxed and only 11.12 ± 1.06% of Te was unconjugated (Figure 2(c)). However, when SHBG was also added, only 4.41 ± 2.1% of Te was glucuronidated and 60.84 ± 1.93% of Te was unconjugated. Following 24 hours in culture with Te, LNCaP cells had glucuronidated and effluxed 67.07 ± 5.48% of Te and only 3.8 ± 1.59% remained unconjugated. Again, less Te was glucuronidated when cells were incubated with Te and SHBG for 24 hours with 13.19 ± 4.93% of Te glucuronidated compared to 67.11 ± 11.74% unconjugated.

### 3.4. Effect of Te and SHBG on LNCaP Cell Growth

**Figure 3:**
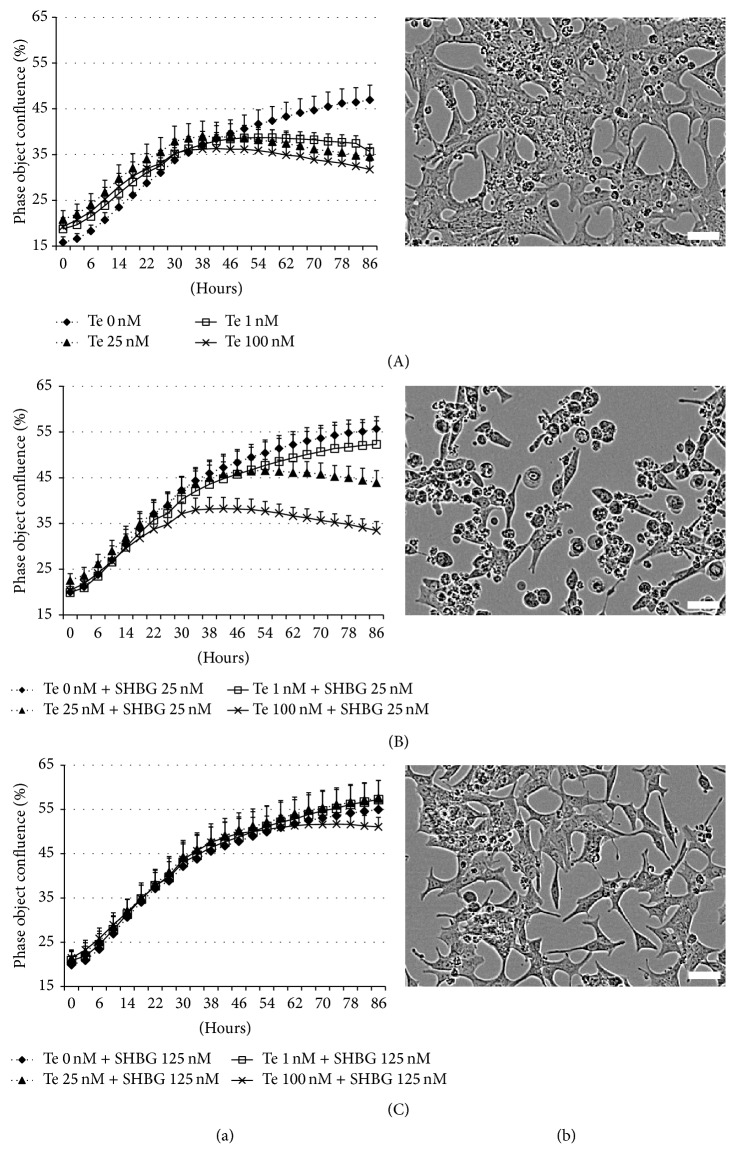
Effects of testosterone and SHBG on LNCaP cell growth and morphology: (a) growth measured using the IncuCyte ZOOM Live Imaging System. LNCaP cells were treated with ((a)(A)) 0, 1, 25, and 100 nM testosterone (Te) in serum-free medium, ((a)(B)) 25 nM Sex Hormone Binding Globulin (SHBG) plus 0, 1, 25, and 100 nM testosterone in serum-free medium, and ((a)(C)) 125 nM SHBG plus 0, 1, 25, and 100 nM testosterone in serum-free medium. Measurements gathered over 86 hours without changing medium. Values are expressed as mean percentage phase object confluence using 9 nonoverlapping images per well. (b) Morphology assessed by IncuCyte Imaging. Cells were imaged after 86 hours in culture using IncuCyte ZOOM Live Imaging System. ((b)(A)) LNCaP cells cultured in serum-free medium were used as the normal control. ((b)(B)) Cells treated with 25 nM testosterone only and ((b)(C)) cells treated with 25 nM testosterone plus 125 nM SHBG. Bar = 50 *μ*m.

LNCaP cells were seeded at initial 20% confluence and after 38 hours of culture cell confluency reached about 35–40% with no differences between treatment groups (Figure 3(a)(A)). Cells grew well in serum-free medium for up to 86 hours. Exposure to only SHBG did not alter cell growth. However after 38 hours in culture, growth declined in a dose dependent manner in those cells treated with 1, 25, and 100 nM Te. Addition of either 25 or 125 nM SHBG canceled the inhibitory effects of Te in a dose dependent fashion (Figures 3(B) and 3(C)). Moreover, cells cultured in Te alone displayed morphological changes in that cells became rounded rather than spread out and detached from the culture plate (Figure 3(b)(B)). Control cells and those incubated with Te plus SHBG did not have these morphological changes (Figures 3(b)(A) and 3(b)(C)).

### 3.5. Effect of SHBG on Te-Sensitive Gene Expression

**Figure 4:**
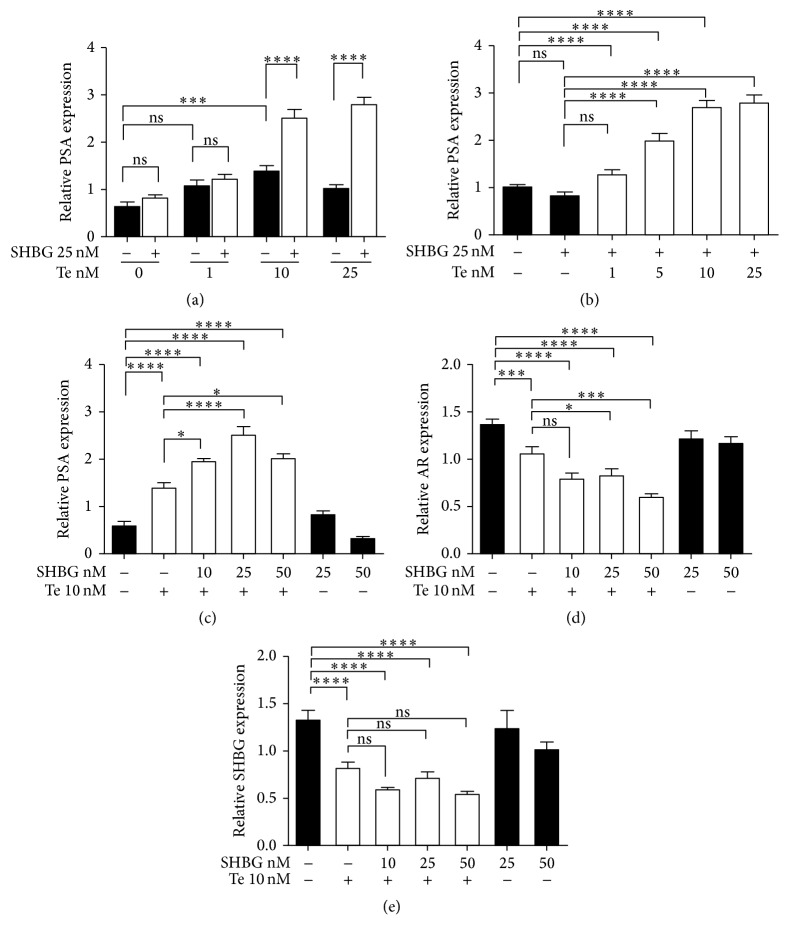
Effects of testosterone (Te) and Sex Hormone Binding Globulin (SHBG) on prostate specific antigen (PSA), androgen receptor (AR), and SHBG mRNA expression in LNCaP cells by quantitative Real-Time PCR. (a) PSA mRNA expression in cells treated with 0, 1, 10, and 25 nM Te ± 25 nM SHBG for 24 hours. (b) PSA mRNA expression in cells treated with 25 nM SHBG plus 0, 1, 5, 10, or 25 nM Te. Expression of PSA (c), AR (d), and SHBG (e) mRNA in LNCaP cells treated with 10 nM Te plus 0, 10, 25, or 50 nM SHBG for 24 hours. All experiments were performed at least three times in triplicate. The mRNA expression was normalised to the housekeeping gene, *β*2-microglobulin (B2M). One-way ANOVA with Tukey's test was used for statistical analysis. Tukey's test indicated as “no significance” (ns) or asterisk (^*∗*^ *P* < 0.05; ^*∗∗∗*^ *P* < 0.001; and ^*∗∗∗∗*^ *P* < 0.0001). All results were represented as mean ± standard error (SEM).

Figure 4(a) displays a paired comparison of treatment of LNCaP cells with 0, 1, 10, and 25 nM Te alone and with the addition of 25 nM of SHBG for 24 hours. Surprisingly, addition of SHBG, despite having previously been shown to reduce Te uptake, significantly enhanced Te upregulation of PSA mRNA expression at Te concentrations of 10 and 25 nM (*P* < 0.0001) (Figure 4(a)). When cells were incubated with 25 nM SHBG plus 0, 1, 5, 10, and 25 nM Te (Figure 4(b)), expression of PSA was increased by Te (*P* < 0.0001). When cells were incubated with 10 nM Te with 0, 10, 25, or 50 nM SHBG, PSA mRNA expression increased with increasing amounts of SHBG to a maximum at 25 nM SHBG (*P* < 0.001) (Figure 4(c)).

In the same samples, androgen receptor (AR) mRNA expression was decreased by Te and expression was further decreased by the addition of 25 or 50 nM SHBG (Figure 4(d)). SHBG mRNA expression was significantly decreased by Te (*P* < 0.0001) (Figure 4(e)). Additionally, SHBG further decreased the Te downregulation of SHBG expression at all SHBG concentrations; however this did not reach statistical significance.

## 4. Discussion

The primary role of hepatically produced SHBG as a serum carrier of sex hormones is well documented [5, 15]. SHBG expression has also been demonstrated in sex hormone target tissues such as human prostate [16, 17], LNCaP cells [18], testes, duodenum, ovary, placenta, proximal tubule epithelial cells (PTEC), and cerebral cortex as well as several cancers [10, 15, 19–21]. Higher expression of SHBG in prostate cancer tissue is associated with poor clinicopathological features suggesting a role in prostate cancer progression [22], perhaps due to prolonged expression of androgen responsive genes [10]. It has also been proposed that locally regulated and produced SHBG may be destined to participate in signalling at the prostate cell membrane [19]. Using a variety of technical approaches we have confirmed that LNCaP cells take up SHBG in a dose dependent but not androgen dependent manner.

It is well known that SHBG reduces cellular uptake of Te; however we have demonstrated that SHBG may also maintain stable levels of biofunctional Te and physiologically relevant intracellular Te levels by reducing Te glucuronidation and efflux. Another study has also demonstrated that LNCaP cells incubated in 30 nM DHT glucuronidated 85% of the DHT in 5 days. Upon addition of 140 nM SHBG, 95% of DHT was not taken up by cells and remained in the medium [23]. This rapid inactivation of Te by glucuronidation has been demonstrated in human clinical studies, where a single oral dose of Te did not alter serum total Te levels but rapidly and significantly increased levels of conjugated Te in serum [24]. However we acknowledge that the level of free Te in healthy men is much lower (174–729 pmol/L [25]) than that used in this study, since* in vivo* SHBG is always present. When SHBG is reduced or absent, excess free Te may enter cells and oversaturate the ARs since it has been reported that AR saturation occurs at approximately 2-3 nM in prostate [26]. The LNCaP cells may respond to this by rapidly glucuronidating and effluxing the excess Te. The steroid content of SHBG (Fitzgerald Industries International) was not specified; however it was purified from the serum of pregnant women which contains significantly lower Te than that of adult males [3, 27]. Even if all of the serum Te were carried over, the Te at the maximum SHBG concentrations used (125 nM) would be much less than 1 nM, significantly less than that used in our experiments. Importantly, SHBG treatment had no significant effect on gene expression when compared to untreated controls.

To further explore the intracellular biological actions of SHBG the effects of SHBG on Te responsive genes were investigated. As expected Te upregulated PSA mRNA expression. However, despite the significantly lower intracellular Te in the presence of SHBG, addition of SHBG in combination with Te further increased the expression of PSA mRNA. Similarly, downregulation of AR mRNA expression by Te was also further enhanced when cells were incubated with SHBG and Te. Addition of SHBG also further decreased the Te downregulation of SHBG mRNA although, under these conditions, the reduction did not reach statistical significance. In this experiment the cellular Te level was lower in the presence of SHBG than when Te was added alone; however the effects on gene expression were much greater. Moreover, maximal effects on gene expression were reached at a lower concentration of Te when SHBG was present. Intracellular expression of SHBG in mouse proximal tubule cells has also been shown to prolong the expression of androgen responsive genes [10] and overexpression of SHBG in LNCaP cells has been shown to influence the expression of estrogen and androgen responsive genes [28]. This would suggest that both endogenously expressed SHBG and internalised SHBG have similar positive stimulatory effects on androgen sensitive gene expression.

In our* in vitro* study, Te decreased SHBG gene expression. A downregulation of serum SHBG also occurs in healthy men following Te administration [29]. We speculate that, in pathological conditions where serum SHBG is reduced, for example, in overweight and obese men [30–33], Te uptake, glucuronidation, and efflux would be increased potentially exacerbating Te deficiency.

In the present study Te treatment did not promote cell growth, and in fact a dose dependent inhibition of growth was demonstrated after 38 hours in culture. Coincubation with SHBG prevented the Te inhibition of cell growth. A biphasic effect on cell proliferation has previously been described where proliferation was increased up to 0.3 nM DHT and then progressively decreased when concentrations were raised above 0.3 nM DHT [23, 34]. The inhibitory effect of DHT was abrogated by addition of human serum [34] or SHBG [23]. The effect of the synthetic androgen methyltrienolone (R1881), which binds poorly to SHBG, was not affected by addition of SHBG [23]. Other synthetic androgens displayed a similar inhibitory effect in LNCaP cells resulting in morphological changes and inhibition of colony formation; however these effects were not reproduced in the androgen receptor-negative prostate cells lines PC-3, DU145, and MRC-5. Furthermore, addition of antiandrogen drugs (6-chloro-6-dehydro-17x-acetoxy-hla, cyproterone acetate, and hydroxyflutamide) restored LNCaP cell growth [35]. All of these studies suggest that inhibition is through an AR pathway. In the present study the striking changes in cell morphology suggest that the cells may be stressed, perhaps due to the requirement to glucuronidate high levels of intracellular Te; however previous studies have not included descriptions of cell morphology in order to make a comparison. The mechanism by which Te inhibits cell growth in LNCaP cells after 38 hours is unknown. Regardless of the percentage of cell confluence at the beginning of treatment, Te always caused morphological changes (data not shown). This perhaps suggests that Te had an effect on cell morphology and attachment rather than directly on cell proliferation. However, the inhibition of cell growth by Te warrants further investigation.

## 5. Conclusions

In this study we have demonstrated that in the absence of SHBG large amounts of Te rapidly enter the cell where they are inactivated by conjugation to glucuronic acid and effluxed. In the presence of SHBG however, while there is reduced Te uptake, glucuronidation and efflux of Te are also reduced and the effects of Te on androgen responsive genes are enhanced. This* in vitro* study of the role of SHBG may have significant clinical relevance, and as such, the assessment of androgen deficiency* in vivo* should simply rely not only on measurements of total Te alone but also on the evaluation of serum SHBG levels.

These findings clearly demonstrate that SHBG plays an important regulatory and intracellular role to modify Te metabolism and function and to promote cell growth. These observations will need to be confirmed in other androgen sensitive tissues. If a consistent effect is demonstrated, it will significantly change our current understanding of the role of SHBG in health and disease.

## Figures and Tables

**Table 1:** Primer sequences for quantitative RT-PCR.

Gene name	Primers	Location	GenBank accession number
PSA	(F) 5′-GTGTGTGGACCTCCATGTTATT-3′ (R) 5′-TGCCCCATGACGTGATACCT-3′	516-537 691-672	NM_001648

AR	(F) 5′-GGCTGTCATTCAGTACTCCTG-3′ (R) 5′-GG AGC CAT CCA AAC TCT TGA-3′	3320-3340 3491-3510	NM_000044

SHBG	(F) 5′-GCCCAGGACAAGAGCCTATC-3′ (R) 5′-CCTTAGGGTTGGTATCCCCATAA-3′	149-168 277-255	NM_001146281

B2M	(F) 5′-GGCTATCCAGCGTACTCCAAA-3′ (R) 5′-CGGCAGGCATACTCATCTTTTT-3′	57-77 302-281	NM_004048

Reference: http://pga.mgh.harvard.edu/primerbank/index.html.

(F): forward; (R): reverse; PSA: prostate specific antigen; AR: androgen receptor; SHBG: sex hormone binding globulin.
